# Using oxygen 18 isotope to problematize the presence of resettled laborers in the far provinces of the Inca empire

**DOI:** 10.1371/journal.pone.0237532

**Published:** 2020-08-10

**Authors:** Francisco Garrido, Catalina Morales

**Affiliations:** Museo Nacional de Historia Natural, Santiago, Chile; Appalachian State University, UNITED STATES

## Abstract

Resettlement, as a major imperial policy in the Inca empire, appears to have been a widespread mechanism for labor mobilization and the dismantling of rebellions. While multiple ethnohistorical references exist regarding resettlement in the central Andes, the extent of this policy in the imperial provinces is still unknown, especially in cases of economic intensification that might have required more labor force. The δ^18^O isotope is a good proxy for human mobility when comparing the childhood isotopic signature in the teeth enamel and the local water signature at the place of death. If applied to the study of an archaeological sequence, we can observe the expansion or reduction of a population’s displacement within a territory, if they received foreigners, and in general, how their social interaction and networks changed over time. In a marginal provincial setting of the Inca empire, such as Copiapó valley in Chile, the study of δ^18^O isotope can enable us to observe if the alleged economic intensification in metallurgical production implied the massive arrival of foreign populations. Significantly, the Late Horizon does not evidence a great change in terms of mobility, compared to previous periods in Copiapó valley. Thus, the isotopic evidence can more clearly illuminate the social and political dynamics of an imperial provincial setting, where economic activities demanded by the Inca state were mainly carried out by the local labor force.

## Introduction

The political economy of the Inca empire was based on prestige and staple goods produced by a massive labor force. This workforce was part of a system of labor tax provided by local populations [1–4]. Drawing from ethno-historical documents, particularly by the chronicler Bernabé Cobo, scholars have conventionally argued that a large part of the conquered populations was mobilized for the labor requirements of the empire, which comprised up to a third of the members of each community in the Andes [5]. These resettled workers known as *mitmaqkunas* traveled long distances to work in the army, agriculture, road construction, mining, and in general, for any infrastructure project where the state required labor [e.g., 6–8]. Resettlement could also have been part of a strategy to deal with difficult populations, in order to avoid rebellions and ensure their loyalty to the state [5]. Their social status in their new settlements was not always the same as that of their places of origin, and sometimes the resettled populations might have been economically marginalized [9].

In general, *mitmaqkunas* were intrusive populations. Their presence implied an important reorganization of labor and modification of the landscapes that they inhabited [10]. The archaeological identification of these resettled groups is complicated, because there is no clear distinction between the introduction of external laborers and the co-optation of local populations. They might also share a similar material culture with the host population [11], and if they have stayed in a place for more than one generation, their bone chemistry might appear identical to that of the local people [10]. Despite the paucity of *mitmaqkuna* presence in the archaeological record, most major changes in terms of the economic intensification of production have been attributed to their presence, or to the general application of the labor tax system. This situation has been largely assumed than demonstrated, particularly in the far provinces of the empire [12].

Since the stylistic study of material culture presents important challenges to the identification of *mitmaqkunas*, archaeometric studies that have dealt with artifact and human provenance have provided relevant insights into human mobility across the Andes during Inca times. For example, in the Puruchuco-Huaquerones cemetery in Lima, Haun and Cock [13] have used biodistance analysis to demonstrate that women had a more diverse provenance than men, were more related to the central Andes than the coast, and likely arrived as craftworkers for the empire. Even at the capital in Cuzco, the DNA evidence reveals that important numbers of the Inca population might have come from the southern Titicaca regions, demonstrating the wide spectrum of people’s movements during that time [14]. Additionally, bone chemistry studies, particularly those using oxygen, strontium, and lead isotopes, have evidenced the presence of long distance migrants in Machu Picchu, who likely arrived as part of the policy of labor mobilization by the Inca state [15]. Neutron activation analyses in pottery have also showed that some of the imperial Inca styles in northern Chile were brought from the southern Titicaca basin. However, their actual link with migrant populations is not yet clear [16]. An exception is the case of Inca pottery from Cuzco and Titicaca regions that was carried to the southern Andes by foreign parties in the context of mountain shrine rituals or capacocha [17,18]. Nevertheless, this case only refers to a specific state ceremony, and does not imply the presence of the *mitmaqkuna* institution in the region.

Although there is some basis to argue for the presence of *mitmaqkuna* colonies in the central Andes, it is still unclear if and to what extent this institutional practice was implemented in the imperial Inca provinces, specifically in the southern cone of the Americas. For example, there are some ethno-historical references for northwestern Argentina [7,19], but we have no such records for the northern Chilean territory. To understand how extensive the *mitmaqkuna* practice was, in this paper, we explore the case of Copiapó valley, which was a marginal provincial setting located at the southern end of the Atacama desert in Chile. As in other parts of northern Chile, important changes took place during the Late Horizon here, such as the intensification of mining activities by the state, particularly copper exploitation [20–22]. Around 1400 AD, Copiapó valley was incorporated into the Inca empire, which transformed its settlement pattern and economic activities. There were new administrative centers built in the Inca architectural style, with plazas and square-shaped buildings [23,24]. The valley was integrated with the rest of the empire by two branches of the Inca Road, one through the desert and the other across the Andes via Argentina [25,26]. The incorporation of new subjects to the empire was a selective process, where some communities experienced more intervention than others. Furthermore, there is some evidence of the use of mechanisms of ideological violence to ensure compliance and avoid rebellions [27].

The few ethnohistorical references available regarding this region do not mention the presence of *mitmaqkunas* or any other foreign group. However, it has been argued that the Inca administration in Copiapó valley was managed by culturally different polities from the southern valleys, known as the Diaguitas [28–30]. This argument is based on the fact that there are no Diaguita pottery styles before the Late Horizon in Copiapó valley, and that such styles only became ubiquitous during the time of the Inca expansion. Nevertheless, it is still unclear if the Diaguita arrived in significant numbers, or if their pottery style had simply spread among local Copiapó people as a prestige good.

In economic terms, it has been traditionally interpreted that one of the main Inca interventions in Copiapó valley was the intensification of mining and metallurgical activities, as evidenced by the large foundry of Viña del Cerro, which comprises of 26 smelters, a plaza, and square-shaped rooms [23,31]. However, it remains unclear what kind of metallurgical production was carried out at this foundry, including its yield, if alloys were produced, or if finished artifacts were manufactured. On the one hand, if Viña del Cerro represents an effort to intensify the regional mining and metallurgical production, it is relevant to question if there was a massive arrival of foreign groups or if most of the labor was carried out by local people. On the other hand, it is also necessary to determine the possible presence of intermediate groups, such as the Diaguita, that might have played an important role in the Inca administration of the territory. Therefore, in this paper, we examine if there was a significant presence of foreign individuals during the Late Horizon, and if that situation differs from previous archaeological periods in Copiapó valley. This analysis is based on oxygen 18 isotopic data from teeth enamel and AMS radiocarbon dating, using 31 samples from the Formative, Middle, Late Intermediate and Late Horizon periods.

## Materials and methods

To understand the impact of human mobility on the archaeological sequence of Copiapó valley, we compared the oxygen isotope data of a sample of 31 individuals that range from the Formative to the Late Horizon periods (Table 1). All sample data was obtained by our team with the exception of individuals 14 and 16 from the Ramadillas site [32]. For the rest of the samples, we obtained radiocarbon dates for all, except for case 23, who shared the burial with individual 24. All of the samples correspond to individuals above 16 years old, with exception of individuals 15 (9 years old), 23 (5 years old), 25 (7 years old) and 26 (13 years old). We included these younger individuals because of the possibility that they could have traveled with their parents from other regions. All the samples came from burials located within the middle and upper course of the Copiapó river. No coastal samples were available for this study. We assigned individuals to period based on their radiocarbon dates and by analyzing the associated material culture. Unfortunately, the Late Intermediate period is the least represented in this sample, with only 3 available samples. Thus, most of our comparisons were done between the Late Horizon and the Middle period. The archaeological human bone samples were obtained from and authorized by public museums in Chile (Museo Regional de Atacama and Museo Nacional de Historia Natural). All necessary permits for sending the samples abroad for radiocarbon and isotopic analyses were issued by the National Council of Monuments (Consejo de Monumentos Nacionales), Ministry of Culture, which complied with all relevant regulations.

**Table 1:** List of the individuals analyzed in this study.

ID	Sample Id (UGAMS lab)	Site	Repository	Tooth enamel sample	Enamel development age (years old)	Period	14C age years, BP	δ^18^Oap (vPDB)	δ^18^O Drinking water value (SMOW) [33]
1	36073	El Torin	MNHN	Premolar	3–8	Formative	1510 +/- 20 (UGAMS 36052)	-5.8	-9.5
2	36074	El Torin	MNHN	Canine	2–7	Formative	1540 +/- 20 (UGAMS 36053)	-2.9	-4.2
3	44086	El Torín	MNHN	Premolar	3–8	Formative	1500 +/- 25	-7	-11.7
4	44087	El Torín	MNHN	Canine	2–7	Formative	1290 +/- 25	-5.4	-8.7
5	36078	Cabra Atada	MNHN	First or second molar (fragment)	2–8	Formative	1430 +/- 25	-10.6	-18.2
6	36077	La Puerta A	MNHN	Premolar	3–8	Formative	1500 +/- 20	-4.9	-7.8
7	36076	La Puerta A	MNHN	Premolar	3–8	Middle	1080 +/- 20	-5.9	-9.7
8	44079	La Puerta A	MNHN	Second molar	4–8	Middle	980 +/- 20	-1.1	-0.9
9	44083	La Puerta A	MNHN	Second molar	4–8	Middle	1040 +/- 20	-3.5	-5.2
10	44082	La Puerta A	MNHN	First molar	2–8	Middle	1000 +/- 20	-6.6	-10.9
11	44080	La Puerta A	MNHN	Second molar	4–8	Middle	1000 +/ 25	-3.8	-5.8
12	44081	La Puerta A	MNHN	First molar	1–3	Middle	940 +/- 20	-6.1	-10
13	36068	Iglesia Colorada	MRA	Premolar	3–8	Late Intermediate	820 +/- 20	-5.7	-9.4
14	29323	Ramadillas	MRA	Second molar	4–8	Late Intermediate	730 +/- 20	-7.4	-12.4
15	36060	Rinconada de San Fernando	MRA	Premolar	3–8	Late Intermediate	640 +/- 20 (UGAMS 36038)	-5.9	-9.7
16	29325	Ramadillas	MRA	Second molar	4–8	Late Horizon	530 +/- 20	-7.6	-12.7
17	36065	Iglesia Colorada	MRA	Incisor	1–5	Late Horizon	630 +/- 25	-6.8	-11.3
18	36067	Iglesia Colorada	MRA	Second molar	4–8	Late Horizon	530 +/- 20	-7.5	-12.6
19	36062	Iglesia Colorada	MRA	First or second molar (fragment)	2–8	Late Horizon	450 +/- 20	-6.7	-11.1
20	36063	Iglesia Colorada	MRA	Second molar	4–8	Late Horizon	420 +/- 20	-6.7	-11.2
21	36064	Iglesia Colorada	MRA	Third molar	10–12	Late Horizon	390 +/- 20	-6	-9.9
22	36066	Iglesia Colorada	MRA	Second molar	4–8	Late Horizon	460 +/- 20	-7.1	-11.8
23	36069	Iglesia Colorada	MRA	Molar in formation	1–7	Late Horizon	NA	-6.2	-10.1
24	36070	Iglesia Colorada	MRA	First or second molar (fragment)	2–8	Late Horizon	500 +/- 25	-0.9	-0.5
25	36071	Iglesia Colorada	MRA	Molar in formation	1–7	Late Horizon	560 +/- 20	-6.1	-9.9
26	36075	Iglesia Colorada	MRA	Premolar	3–8	Late Horizon	490 +/- 25	-4.3	-6.7
27	36072	Iglesia Colorada	MRA	Canine	2–7	Late Horizon	530 +/- 20 (UGAMS 36051)	-8.1	-13.8
28	44091	Rinconada de San Fernando	MRA	Premolar	3–8	Late Horizon	520 +/- 30	-6.4	-10.6
29	36059	Rinconada de San Fernando	MRA	Second molar	4–8	Late Horizon	560 +/- 20 (UGAMS 36037)	-6.6	-10.8
30	36058	Punta Brava summit	MRA	First or second molar (fragment)	2–8	Late Horizon	480 +/- 20 (UGAMS 36036)	-6.1	-10
31	36061	Copiapó downtown	MRA	Second molar	4–8	Late Horizon	350 +/- 20 (UGAMS 36039)	-6.2	-10.2

In the column repository, the abbreviations are as follows: MNHN (Museo Nacional de Historia Natural, Santiago); MRA (Museo Regional de Atacama, Copiapó).

There is great potential for the use of δ^18^O stable isotope to contribute to the study of human mobility, because its value is related to the specific sources of water that a person consumed during childhood. Oxygen isotope values in human body tissue are proportionally related to the composition of drinking water, and to a lesser extent, oxygen in air and food sources. The δ^18^O in body water equilibrates with the δ^18^O in hydroxyapatite during its formation, and dental enamel isotope values become fixed during the permanent teeth development stage [33–39]. Considering that the isotopic composition of ground water varies according to latitude, altitude, aridity, seasonal temperature change, and rainfall, those variations make δ^18^O a relevant geographical marker for the assessment of people’s mobility [35,40–42]. Nevertheless, a proper interpretation of the relationship between isotope values and human mobility is not possible without an adequate knowledge of the variation and distribution of water sources in the area under study.

For this research, we obtained the δ^18^O values and AMS dates from dental enamel bioapatite carbonate (Table 1). In some cases, the radiocarbon dates were obtained from collagen from other bones of the same individual. All the samples were analyzed at the Center for Applied Isotope Studies (CAIS) from the University of Georgia, which uses a NEC 500KV tandem Pelletron accelerator achieving precisions of 0.35% for radiocarbon dates. For the specific measurement of the stable isotope ratios of oxygen, CAIS uses a Thermo 253 Isotope Ratio Mass Spectrometer. To estimate the geographic relationship between the individuals from Copiapó valley, the original VPDB δ^18^O dental enamel carbonate values were converted into SMOW values by using the formula 1.03092*(VPDB value)+30.92. The next step, which is to correlate the enamel carbonate signature with water sources can generate an important error ranging from 1 to 3.5‰ in the δ^18^O isotope value, depending on what formula is used as determined by Pollard [43]. We used the formula by Chenery et. al. [33] because it works directly with enamel carbonate values with an error estimate of 1‰ (95% CI), which is at the lower end of the estimate given by Pollard [42]. Most of the other formulas use δ^18^O phosphate values, which would require an extra step for the conversion of our data. Thus, our data was converted into “drinking water” values by using the following formula: δ^18^O DW = 1.786* δ^18^Ocap– 54.005 [33]. We then compared the location of the sample individuals with the hydrological isoscape of Copiapó valley.

### Oxygen 18 isoscape in Copiapó valley

The δ^18^O stable isotope analysis is useful in hydrological studies to determine mechanisms of evaporation and recharge in a watershed [35,43]. In most of the western slope of the southern Andes there is a common pattern of increasing of δ^18^O values when approaching the Pacific Ocean coast, a situation that is also affected by the latitudinal difference in precipitations [42]. Rain and snow occur generally at high altitudes, refilling the rivers and underground water sources. To correlate our data with a geographical origin, we used 160 georeferenced water sample points from Copiapó river, obtained from a survey commissioned by the National Geological Service (Sernageomin) between 1996 to 2010 [44,45]. Collected data included surface and groundwater. The main source of water in Copiapó comes from snow with very low δ^18^O values at high mountain elevations. These values increase as altitude declines due to evaporation on its way downstream [46]. In the Andes and other regions of the world, the wide variety of water sources, the differential amount of precipitation, and cultural practices of alcoholic beverage consumption can be problematic for a clear distinction of δ^18^O regions [35,47,48]. However, in the case of Copiapó river, its location within the Atacama desert makes rainfall almost nonexistent. In this regard, the lack of lower altitude precipitations makes the oxygen isotopic values more stable, with a gradual east-west variation from the mountains to the coast. The estimation of the provenance of human remains is more reliable under these conditions, since Copiapó river’s δ^18^O values are not affected by seasonal variation in precipitation, and all the water comes from a common source in the mountains.

**Fig 1:**
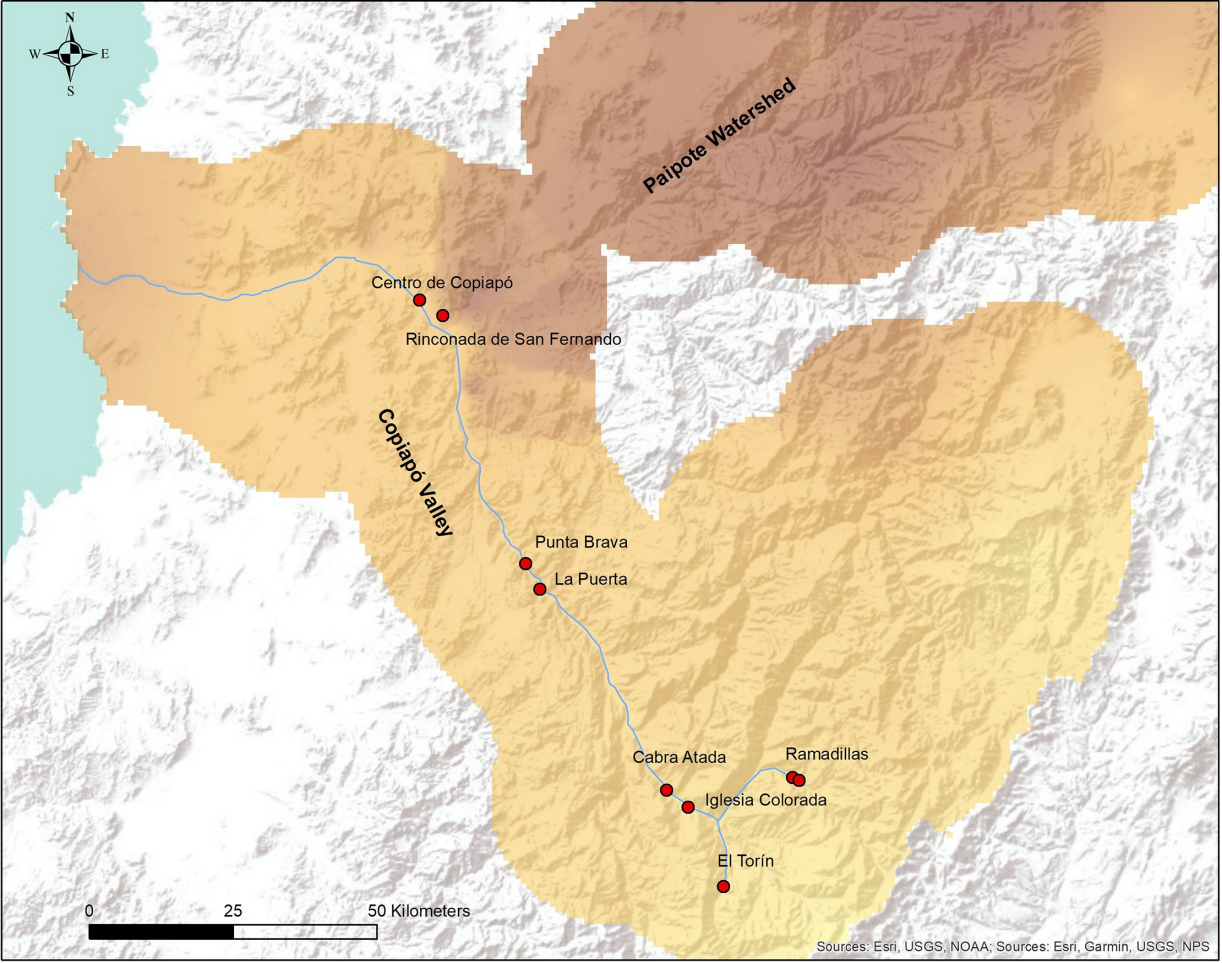
Copiapó valley isoscape. It includes the location of the main archaeological sites cited in the study.

We used Copiapó river isotopic data to create an interpolated surface raster of one square kilometer per pixel using empirical Bayesian kriging in ArcGis 10.5 software. This method of interpolation uses the principle of spatial autocorrelation to model spatial patterns, but more importantly, it is capable of measuring the statistical uncertainty associated with a predicted value. Moreover, the empirical Bayesian kriging does not assume a tendency toward an overall mean, including an intrinsic random function that corrects for trends in the data [49,50]. This interpolation corresponds to the expected local isotopic water values for each location in the valley, creating a δ^18^O continuous isoscape of the region (Fig 1). We also added two other layers that reflect the standard error of the Bayesian kriging at a 95% confidence level, considering the upper and lower ranges of that estimation. We then extracted δ^18^O values from the interpolated surface every 1 kilometer from the coast to the mountains within Copiapó valley, taking into consideration the previously obtained upper and lower standard error. This data formed the basis for the analysis of provenance, comparing these results with the isotope values from the human samples, to determine if they are likely or not to have come from Copiapó valley.

## Results

We analyzed our data in two different ways. First, we examined the internal spread of δ^18^O values per period, and second, we compared our sample with the local isotopic values from Copiapó valley. These allowed us to observe if the Late Horizon evidenced any significant changes compared to previous periods, and if there were individuals who did not match the local isotopic signal. In general, two main patterns emerged. On the one hand, there was no constant variation in terms of mobility through time, and on the other, the Late Horizon did not correlate with a higher increase in the presence of foreign population.

**Fig 2:**
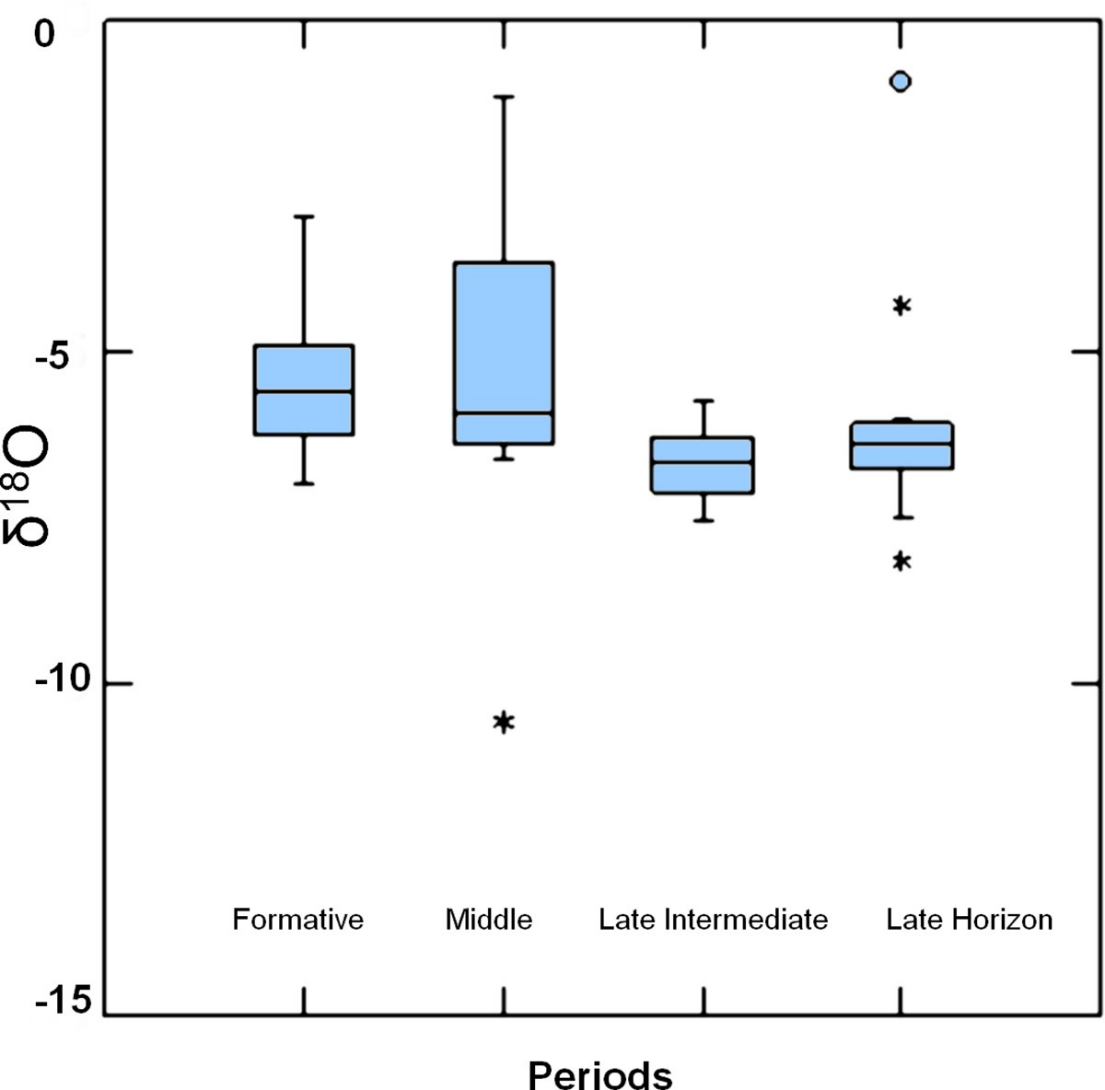
δ^18^O isotope values by period. Box plot graph showing the distribution of δ^18^O isotope values by period in Copiapó valley.

In the box plot (Fig 2), we observe the distribution and spread of δ^18^O values per period. The graph shows that the data splits in two groups: one that comprises the Formative and Middle period, and a second one that includes the Late Intermediate period and Late Horizon. This means that the provenance of people buried during the Formative and Middle periods came from a wider range of locations than the Late Horizon. The Middle period represents the larger spread of δ^18^O isotope values, where after that, there is a reduction in the diversity of people’s provenance.

The decrease in human mobility during the Late Horizon likely reflects some changes in previous long-distance networks, which constrained people’s movements within the valley. This might be part of a pattern starting from the Late Intermediate period. From the 16 analyzed individuals of the Late Horizon, most people seemed to share a similar isotopic signature, except for one far outlier that might signal the presence of foreign arrivals.

**Fig 3:**
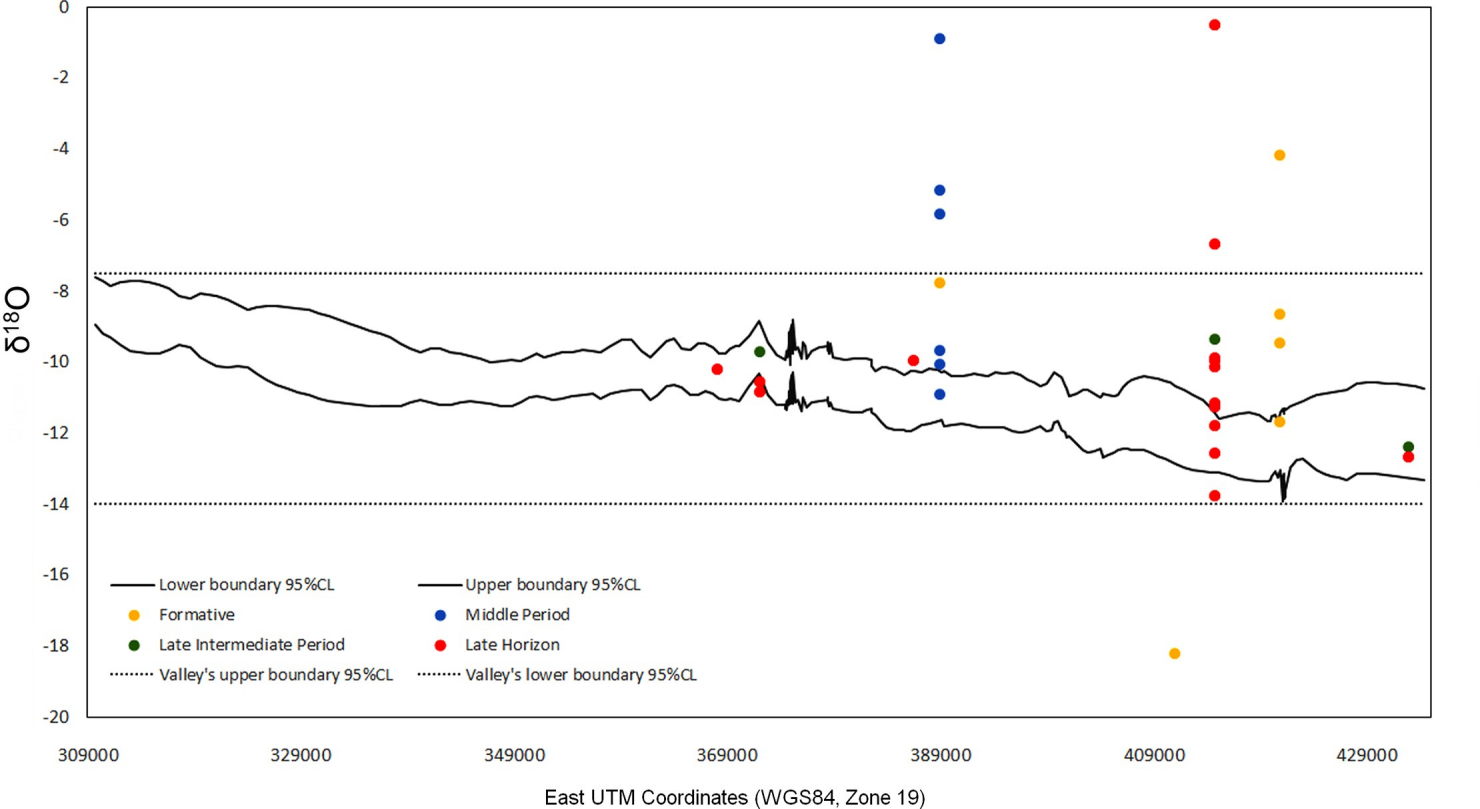
Geographic dispersion of δ^18^O values in Copiapó valley. East-west dispersion of δ^18^O values of prehispanic individuals from Copiapó valley (points), compared to the water values at 95% confidence level (solid lines). The straight dotted lines represent the maximum and minimum δ^18^O values considering the total valley area. The X axis represents east UTM coordinates (WGS 84, zone 19).

To compare our data with the local isoscape, we created a graph that shows the profile of δ^18^O values from Copiapó valley (Fig 3). Data for the graph was taken from the interpolated isoscape that we created, extracting δ^18^O values every kilometer in a west-east direction to represent the valley extending from the coast to the Andes mountains. The graph was plotted with two parallel lines that form a “tunnel”, representing the upper and lower error range of δ^18^O values at 95% confidence level. Above and below this “tunnel” are two dotted straight lines that represent the maximum and minimum range of δ^18^O values in Copiapó valley. In between the lines, we plotted the tooth enamel values by period as points, converted into SMOW or “drinking water” values by using Chenery et al.’s formula [33].

The data for the Formative period reveals that people moved across the lower and upper valleys, since their δ^18^O values from childhood do not coincide with the local water of their places of burial. Considering that in this region, this is the period of transition from hunter gathering to agriculture, it was likely that there were more traffic circuits as compared to more consolidated agriculturalist societies. However, the Middle period does not show a reduction in mobility, but on the contrary, a wider distribution of δ^18^O values. There are even two individuals that differ from the local regional values, one higher and one lower in δ^18^O. That data suggests that the people buried in the main mound cemetery of this time, La Puerta, came from various places in the surrounding territory, signaling the presence of communities in the desert outside the valley. In contrast, the Late Horizon and the small sample that we have for the Late Intermediate period show a change in that regard, where people tended to coincide more frequently with the local water values.

The graph evidences that for most individuals from the Late Horizon, their locations of childhood and death correspond to Copiapó valley. There is only one individual from the Late Horizon that falls completely outside of the regional isotopic values; this person might have come from either northwestern Argentina or farther north because of the higher δ^18^O values, which are related to a climate with more water evaporation. Interestingly, the wider dispersion of δ^18^O values in the Late Horizon happens at Iglesia Colorada, a site located at the upper course of the river, while individuals from the middle course matched better with the expected values for their geographical location. These results make sense considering that Iglesia Colorada is the largest Inca settlement in the valley and is located near to the routes that connect with Argentina [23,27].

## Discussion

During the archaeological sequence in Copiapó valley, two main patterns are visible in terms of mobility: 1) a reduction in provenance variation after the Middle period, and 2) a low presence of the foreign population in nearly all of the sequence analyzed. This illuminates some of the extent of Inca influence in Copiapó valley, considering that the Late Horizon does not evidence relevant changes compared to the rest of the archaeological sequence of this region. Regarding the characteristics of pre-Inca times in Copiapó, during the Formative period known as the “Molle Culture” (circa 2000 BP), the first villages associated with the transition from a hunter gatherer economy to early agriculture are present, including the first evidence of pottery in the region [51]. Most sites are composed of scattered clusters of circular stone structures that possibly reflect the presence of a few extended households. Their burial pattern consisted of funerary mounds filled with dirt and stones, generally located near dwelling areas. As an identity marker, the use of lip piercing or “tembetá” was widespread, just as smoking pipes were used for ritual practices [52]. Around 1500 BP, there is a change in material culture that gives rise to the Middle period, which is characterized by the “Animas Culture”. Here we can see the introduction of painted pottery, rock art paintings, and the disappearance of the tembetá and smoking pipes [53]. The practice of burial mounds continued, but they were concentrated at particular cemeteries that were large, possibly reflecting the presence of supralocal communities. Both periods indicate a wide range of people’s provenance, which is possibly related to the dispersal in the Atacama desert of small-scale, household-based mining camps, which produced turquoise beads and red iron oxide pigments for exchange [25]. This phenomenon can be also explained by the establishment of larger social networks with neighboring regions in Chile and northwestern Argentina, as suggested by the presence of foreign metal artifacts and pottery styles such as Aguada and Sanagasta [54,55]. Because of the remoteness of this region, there was no influence from Tiwanaku, but there was certainly a common contact with Argentina, as evidenced in the local material culture.

In contrast, the Late Horizon shows a reduction in human mobility, which probably started during the Late Intermediate period. The Late Intermediate period between 800–600 BP represents the development of Copiapó culture with a distinctive black on red pottery decoration style. Apart from a change in material culture, the practice of mound burials disappears during this period, and there is presence of at least two small hilltop fortifications, Punta Brava and Manflas, which follow a common pattern of conflict and territoriality in the Andes [23,56–59]. At the same time, the small villages comprising of clusters of circular stone structures remained the same since the beginning of sedentary life in the valley, and there is no evidence of a hierarchical settlement pattern. This coincides with the existence of a decentralized political power as witnessed in the Spanish chronicles from the 16th century [60]. As mentioned earlier, during the Late Horizon the Incas intervened in some local villages, but the main evidence for economic intervention is the expansion of metallurgical activities, as suggested by the Viña del Cerro foundry and the variety of Inca style bronze artifacts that have been found in the region [23,24,61,62]. Although more data is needed regarding the specific labor mechanisms used by the Incas to intensify mining and metallurgical production, the mobilization of large amounts of foreign population is unlikely in this scenario.

Finally, it is important to consider some possible cultural factors that can affect the accuracy of the δ^18^O measurements. Breast-feeding can increase the δ^18^O values in teeth enamel [39,63], affecting the first stages of permanent teeth development. In our sample, the cases in which enamel formation is under 2–3 years old, might have somewhat higher δ^18^O values due to that factor; however, only part of the teeth enamel is formed during the breast-feeding period. After weaning, the δ^18^O value decreases, and averages out with the consumption of solid food and local drinking water [39,64]. Cooking and fermenting practices can also affect water isotope values, increasing individuals’ δ^18^O values [35,65]. The brewing of the fermented beverage known as *Chicha* was commonplace in the Andes during the Late Horizon, and its consumption may affect the oxygen isotopic signal of people [66]. However, this would only affect the teeth enamel if *chicha* was consumed during childhood, which is unlikely. Since Late Horizon individuals in Copiapó do not show higher δ^18^O values when compared to those of previous periods, *chicha* consumption might have been moderate or only relevant to the working adult population during the time of the Inca occupation. Inter-laboratory sample treatment procedures can also produce significant variability of results in the δ^18^O measurements of hydroxyapatite [67]. In our case, we sought to reduce that source of error by analyzing all of our samples in a single laboratory in the University of Georgia.

## Conclusion

Did the Incas introduce large numbers of foreign population to Copiapó valley as administrators or workers? The use of oxygen 18 isotope data suggests that there was no significant presence of foreign populations in practically all of the analyzed archaeological sequence of Copiapó valley. This leads us to propose that the social and economic transformations introduced by the Incas in this region relied mainly on tribute from the local population, and the Incas did not employ the strategy of resettling large populations of workers from external communities. Although our sample is small, a wider dispersion of δ^18^O values would have been necessary to argue for a significant number of foreign arrivals and presence during the Late Horizon.

In general, the evidence suggests that in Copiapó valley, people might have reduced their inter-regional interaction and movement at some point after the Middle period, possibly due to the larger changes of decentralization and conflict experienced in the Central Andes during the Late Intermediate Period. The arrival of the Inca Empire did not mark a big change to this tendency, which indicates that the transformations in settlement pattern and intensification in mining mainly involved the local population in the valley. Another possible interpretation of this study’s findings is that the Incas had brought in seasonal laborers who then returned to their main residences after completing their work period. However, we believe that this scenario is unlikely because we would expect, in this case, to find a higher degree of foreign material culture in the local sites where such seasonal laborers resided. Furthermore, the distance between Copiapó and other populated places might not have favored short-term stays, so if there were large numbers of foreigners, their presence would have been noticeable in the settlement pattern and artifact styles. Regarding the question of whether the *mitmaqkuna* system was ever used in Copiapó, our data only demonstrates that there is no evidence of the significant arrival of foreign individuals. Our analysis does not permit us to determine if people migrated from Copiapó to work elsewhere in the empire. In future research, we expect to analyze larger samples and explore other isotope signals, such as strontium, in order to obtain independent evidence for people’s provenance.

On a broader scale, this study’s findings illuminate some mechanisms that articulated the political economy of the Inca empire, demonstrating the limitations of state power in the provinces to enforce more powerful means of labor reorganization. At the same time, the use of the scarce local labor to intensify mining production might have limited the output of these activities, influencing particular dynamics of coercion and local agency, which contributed to the creation of either more negotiated or violent political relations in the imperial margins [12,27]. More studies in different provincial territories would enable us to better understand how extensive the Inca resettlement policy actually was outside of the imperial core, with all the political and economic consequences that this implies.

## Supporting information

S1 File (DOCX)

## References

[1] D’Altroy TN, Earle T. Staple Finance, Wealth finance, and storage in the Inka political economy. Current Anthropology 1985;26(2):187–206.

[2] La Lone D. The Inca as a non-market economy: supply on command versus supply and demand. In: Ericson J, Earle T, editors. Contexts for prehistoric exchange. New York: Academic Press; 1982 pp. 291–316.

[3] Julien C. The Chincaysusyu road and the definition of an Inca imperial landscape. In: Alcock S, Bodel J, Talbert R. Highways, byways, and road systems in the pre-modern world. Malden: Wiley & Sons; 2012 pp.147–67.

[4] McEwan G. The Incas: new perspectives. Santa Barbara: ABC-CLIO, Inc; 2006.

[5] D’Altroy TN. The Incas. Second edition. Malden: Blackwell Publishing; 2015.

[6] Costin C. Craft production and mobilization strategies in the Inka empire. In: Wailes B, editor. Craft specialization and social evolution. Philadelphia: The university museum of archaeology and anthropology, University of Pennsylvania; 1996 pp.211–55.

[7] Lorandi A, Cremonte B. Evidencias en torno a los mitmaqkuna incaicos en el noroeste argentino. Anthropologica 1991; 9:211–45.

[8] Rostorowski M. Historia del Tahuantinsuyu. Lima: Instituto De Estudios Peruanos; 2008.

[9] Hu D, Shackley MS. ED-XRF analysis of obsidian artifacts from Yanawilka, a settlement of transplanted laborers (mitmaqkuna), and implications for Inca imperialism. Journal of Archaeological Science: Reports 2018;18: 213–221.

[10] Hu D. Making space under the Inca: a space syntax analysis of a mitmaq settlement in Vilcas Huamán province, Peru. Antiquity 2019;93(370): 990–1008.

[11] Covey RA. Inka imperial intentions and archaeological realities in the peruvian highlands. In: Shimada I, editor. The Inka empire: a multidisciplinary approach. Austin: University of Texas Press; 2015 pp.83–96.

[12] Garrido F, Salazar D. Imperial expansion and local agency: a case study of labor organization under Inca rule. American Anthropologist 2017; 119(4): 631–44.

[13] Haun SJ, Cock GA. Bioarchaeological approach to the search for mitmaqkuna. In: Malpass M, Alconini S, editors. Distant Provinces in the Inka empire: toward a deeper understanding of Inka imperialism. Iowa City: University of Iowa Press; 2010 pp. 193–220.

[14] Shinoda K. Tracing the origin of Inka people through ancient DNA analysis. In: Shimada I, editor. The Inka empire: a multidisciplinary perspective. University of Texas Press, Austin; 2015 pp.55–66.

[15] Turner B, Kamenov GD, Kingston JD, Armelagos GJ. Insights into immigration and social class at Machu Picchu, Peru based on oxygen, strontium, and lead isotopic analysis. Journal of Archaeological Science 2009; 36:317–332.

[16] Williams V, Santoro C, Speakman RJ, Glascock MD, Romero A, Valenzuela D, et al Instrumental neutron activation analysis of Inka and local pottery from northern Chile's Atacama Desert. Journal of Archaeological Science Reports 2016; 9:481–492.

[17] Bray T, Minc LD, Ceruti M, Chávez J, Perea R, Reinhard J. A compositional analysis of pottery vessels associated with the Inca ritual of capacocha. Journal of Anthropological Archaeology 2005; 24: 82–100.

[18] Wilson AS, Taylor T, Ceruti M, Chavez J, Reinhard J, Grimes V, et al Stable isotope and DNA evidence for ritual sequences in Inca child sacrifice. Proceedings of the National Academy of Sciences 2007; 104(42):16456–16461. 10.1073/pnas.0704276104 PMC2034262 17923675

[19] Raffino R, Vitry C, Gobbo D. Inkas y Chichas: identidad, transformación y una cuestión fronteriza. Boletín De Arqueología PUCP 2004; 8:247–265.

[20] Cantarutti G. Mining under Inca rule in north-central Chile: the Los Infieles mining complex. In: Tripcevich N, Vaughn K, editors. Mining and quarrying in the ancient Andes. New York, Heidelberg, Dordrecht, London: Springer; 2013 pp. 185–212.

[21] Salazar D, Berenguer J, Vega G. Paisajes minero-metalúrgicos Incaicos en Atacama y el altiplano sur de Tarapacá (norte de Chile). Chungara 2013; 45(1): 83–103.

[22] Zori C, Tropper P, Scott D. Copper Production in late prehispanic northern Chile. Journal of Archaeological Science 2013; 40(2): 1165–75.

[23] Castillo G. Los períodos intermedio tardío y tardío: desde la cultura Copiapó al dominio Inca. In: Cervellino M, Castillo G, Niemeyer H, editors. Culturas pehistóricas de Copiapó. Copiapó: Museo Regional de Atacama; 1998 pp. 163–282.

[24] Niemeyer H. Estrategia del dominio inca en el valle de Copiapó. In: Sociedad Chilena de Arqueología, editor. Proceedings of the XII congreso nacional de arqueología chilena. Temuco: Sociedad Chilena de Arqueología; 1993 pp. 333–371.

[25] Garrido F. Rethinking imperial infrastructure: a bottom-up perspective on the Inca road. Journal of Anthropological Archaeology 2016; 43: 94–109.

[26] Iribarren J, Bergholz H. El camino del Inca en un sector del norte chico. In: Sociedad Chilena de Arqueología, editor. Proceedings of the VI congreso nacional de arqueología chilena. Santiago: Sociedad Chilena de Arqueología; 1972 pp. 229–266.

[27] Garrido F, Morales C. Displays of violence and power at the edge of the empire: provincial trophy heads during Inca times. Latin American Antiquity 2019; 30(3):606–623.

[28] Berenguer J. Chile bajo el imperio de los Inkas. Santiago: Museo Chileno de Arte Precolombino; 2009.

[29] Cornejo L. Los Inka y sus aliados Diaguita en el extremo austral del Tawantinsuyu. In: Museo Chileno de Arte Precolombino, editor. Tras la huella del Inka en Chile, Santiago: Museo Chileno de Arte Precolombino; 2001 pp.74–89.

[30] Uribe M, Sánchez R. Los Incas en Chile. aportes de la arqueología chilena a la historia del Tawantinsuyu. In: Falabella F, Uribe M, Sanhueza L, Aldunate C, Hidalgo J, editors. Prehistoria en Chile. Desde sus primeros habitantes hasta los Incas Santiago; 2016 pp. 529–572.

[31] Niemeyer H. La ocupación inkaica de la cuenca alta del Río Copiapó. Revista Comechingonia 1986; 4:165–294.

[32] Diaz P, Pacheco A. Informe de las dataciones radiocarbónicas y de los análisis de isótopos estables (13C, 15N y 18O) de los contextos funerarios del proyecto Caserones. Santiago: Proyecto Caserones; 2017.

[33] Chenery C, Pashley V, Lamb A, Sloane H, Evans J. The oxygen isotope relationship between the phosphate and structural carbonate fractions of human bioapatite. Mass Spectrometry 2012; 26(3):309–319. 10.1002/rcm.5331 22223318

[34] Daux V, Lécuyer C, Héran MA, Amiot R, Simon L, Fourel F, et al Oxygen isotope fractionation between human phosphate and water revisited. Journal of Human Evolution 2008; 55:1138–1147. 10.1016/j.jhevol.2008.06.006 18721999

[35] Knudson K. Oxygen isotope analysis in a land of environmental extremes: the complexities of isotopic work in the Andes. International Journal of Osteoarchaeology 2009; 19(2): 171–191.

[36] Longinelli A. Oxygen isotopes in mammal bone phosphate: a new tool for paleohydrological and paleoclimatological research? Geochimica et Cosmochimica Acta 1984; 48(2):385–390.

[37] Luz B, Kolodny Y, Horowitz M. Fractionation of oxygen isotopes between mammalian bone phosphate and environmental drinking water. Geochimica et Cosmochimica 1984; 48(8):1689–1693.

[38] Pellegrini M, Lee-Thorp JA, Donahue RE. Exploring the variation of the δ18Oc and δ18Op relationship in enamel increments. Palaeogeography, Palaeoclimatology, Palaeoecology 2011; 310:71–83.

[39] Katzenberg M. A., Waters‐Rist A. Stable isotope analysis: a tool for studying past diet, demography, and life history. In: Katzenberg M.A., Grauer A.L. Biological anthropology of the human skeleton. Hoboken: John Wiley & Sons, Inc.; 2019 pp. 469–504.

[40] Ugan A, Neme G, Gil A, Coltrain J, Tykot R, Novellino P. Geographic variation in bone carbonate and water δ18O values in Mendoza, Argentina and their relationship to prehistoric economy and settlement. Journal of Archaeological Science 2012; 39:2752–2763.

[41] Turner T, Collyer M, Krabbenhoft T. A general hypothesis-testing framework for stable isotope ratios in ecological studies. Ecology 2010; 91(8):2227–2233. 10.1890/09-1454.1 20836444

[42] Gil A, Neme G, Ugan A, Tykot R. Oxygen isotopes and human residential mobility in central western Argentina. International Journal of Osteoarchaeology 2014; 24(1), 31–41.

[43] Pollard M, Pellegrini M, Lee-Thorp JA. Technical note: some observations on the conversion of dental enamel δ18Op Values to δ18Ow to determine human mobility. American Journal of Physical Anthropology 2011; 145:499–504. 10.1002/ajpa.21524 21541927

[44] Aguirre I, Hauser A, Espejo C, Schwarz F. Hidroquímica en el valle del río Copiapó, III región, Chile. In: Sociedad Geológica de Chile, editor. Congreso Geológico Chileno n° 8. Antofagasta: Sociedad Geológica de Chile; 1997 pp. 610–614.

[45] Troncoso R, Espinoza M, Pérez Y, Castro R, Lorca M, Vega N, et al Evaluación hidrogeológica de la cuenca del río Copiapó con énfasis en la cuantificación, dinámica y calidad química de recursos hídricos superficiales y subterráneos. Copiapó: Sernageomin; 2012.

[46] Aguirre I, Hauser A, Schwardtfeger B. Estudio hidrogeológico del valle del río Copiapó, segmento embalse Lautaro–Piedra Colgada, región de Atacama. Santiago: Sernageomin; 1999.

[47] Hermes T, Pederzani S, Makarewicz C. Ahead of the curve? Implications for isolating vertical transhumance in seasonal montane environments using sequential oxygen isotope analyses of tooth enamel. In: Ventresca Miller A., Makarewicz C., editors. Isotopic investigations of pastoralism in prehistory. London and New York: Routledge; 2018 pp. 57–76.

[48] Ventresca Miller A. Modeling modern surface water ⸹18O to explore prehistoric human mobility. In: Ventresca Miller A, Makarewicz C, editors. Isotopic investigations of pastoralism in prehistory. London and New York: Routledge; 2018 pp. 41–55.

[49] Krivoruchko K, Gribov A. Pragmatic bayesian kriging for non-stationary and moderately non-gaussian data. In: Pardo-Igúzquiza E, Guardiola-Albert C, Heredia J, Moreno-Merino L, Durán J, Vargas-Guzmán J, editors. Mathematics of planet earth. Lecture notes in earth system sciences. Berlin, Heidelberg: Springer; 2014 pp. 61–64.

[50] Pilz J, Spöck G. Why do we need and how should we implement bayesian kriging methods. Stochastic Environmental Research and Risk Assessment 2007; 22 (5): 621–632.

[51] Niemeyer H. El periodo temprano del horizonte agroalfarero en Copiapó. In: Cervellino M, Castillo G, Niemeyer H., editors. Culturas prehistóricas de Copiapó. Copiapó: Museo Regional de Atacama; 1998 pp. 61–114.

[52] Troncoso A, Pavlovic D. Historia, saberes y prácticas: un ensayo sobre el desarrollo de las comunidades alfareras del norte semiárido Chileno. Revista Chilena de Antropología 2013; 27(1): 101–140.

[53] Niemeyer H. 1998. El período medio, Complejo las Ánimas. In: Cervellino M, Castillo G, Niemeyer H. Culturas Prehistóricas de Copiapó. Copiapó: Museo Regional de Atacama; 1998 pp.115–162.

[54] Castillo G, Niemeyer H, Cervellino M. Indicadores, alcances y perspectivas de influencias Aguada en el valle de Copiapó. Shincal 1996; 6: 193–212.

[55] Niemeyer H. Pasos cordilleranos y contactos entre los pueblos del norte chico de Chile y el noroeste argentino. In: Nuñez L, Niemeyer H, Falabella F. La cordillera de los Andes, ruta de encuentros. Santiago: Museo Chileno de Arte Precolombino; 1994 pp.23–37.

[56] Arkush E. Hillforts of the ancient Andes. Colla warfare, society, and landscape. Gainesville: University Press of Florida; 2015.

[57] Arkush E, Stanish C. Interpreting conflict in the ancient andes: implications for the archaeology of warfare. Current Anthropology 2005; 46(1): 3–28.

[58] Nielsen A. El estudio de la guerra en la arqueología sur-andina. Corpus 2015; 5(1): 1–9.

[59] Topic J, Topic T. The Archaeological investigation of andean militarism: some cautionary observations. In: Haas J, Pozorski S, Pozorski T, editors. The origins and development of the Andean state. New York: Cambridge University Press; 1987 pp. 47–55.

[60] Garrido F, González S. Adaptive strategies during times of conflict and transformation: copiapó valley under the spanish conquest in the sixteenth century. Ethnohistory 2020; 67(1):127–148.

[61] Garrido F, Li T. A handheld XRF study of Late Horizon metal artifacts: implications for technological choices and political intervention in Copiapó, northern Chile. Archaeological and Anthropological Sciences 2017; 9: 935–942.

[62] Zori C. Extracting insights from prehistoric andean metallurgy: political organization, interregional connections, and ritual meanings. Journal of Archaeological Research 2019; 27: 501–556.

[63] Wright LE, Schwarcz HP. Stable carbon and oxygen isotopes in human tooth enamel: identifying breastfeeding and weaning in prehistory. American Journal of Physical Anthropology 1988; 6:1–18. 10.1002/(SICI)1096-8644(199805)106:1<1::AID-AJPA1>3.0.CO;2-W 9590521

[64] Britton K, Fuller BT, Tütken T, Mays S, Richards MP. Oxygen isotope analysis of human bone phosphate evidences weaning age in archaeological populations. American Journal of Physical Anthropology 2015; 157(2): 226–241. 10.1002/ajpa.22704 25677569

[65] Brettell R, Montgomery J, Evans J. Brewing and stewing: the effect of culturally mediated behaviour on the oxygen isotope composition of ingested fluids and the implications for human provenance Studies. Journal of Analytical Atomic Spectrometry 2012; 27: 778–785.

[66] Gagnon CM, Andrus CFT, Ida J, Richardson N. Local water source variation and experimental chicha de maíz brewing: implications for interpreting human hydroxyapatite δ18O values in the Andes. Journal of Archaeological Science: Reports 2015; 4: 174–181.

[67] Pestle WJ, Crowley BE, Weirauch MT. Quantifying Inter-Laboratory Variability in Stable Isotope Analysis of Ancient Skeletal Remains. PLOS ONE 9 2014; 7: e102844 10.1371/journal.pone.0102844 25061843 PMC4111498

