# Supplementary material for: Using oxygen 18 isotope to problematize the presence of resettled laborers in the far provinces of the Inca empire
Source: PLoS One. 2020 Aug 10;15(8):e0237532. doi: 10.1371/journal.pone.0237532 (PMC7416952; doi:10.1371/journal.pone.0237532)
Supplement: S1 File — (DOCX) [file pone.0237532.s001.docx]

Table with the water sampling data used for the 18O isoscape interpolation

| **Sample ID** | **Origin** | **UTM _E (WGS 84)** | **UTM _N (WGS 84)** | **Altitude** | **18O SMOW** | **Sampling date** | **Reference** |
| --- | --- | --- | --- | --- | --- | --- | --- |
| CPO-CE-064 | Surface water | 308983 | 6977378 | 13 | -8.0 | Spring 2010 | Troncoso 2012 |
| CPO-RC-34 | Surface water | 310775 | 6975810 | 13 | -8.6 | Summer 2009 | Troncoso 2012 |
| CPO-RC-35 | Surface water | 317942 | 6976431 | 64 | -8.8 | Summer 2009 | Troncoso 2012 |
| CPO-RT-47 | Well | 324089 | 6975110 | 104 | -9.3 | 10/13/2009 | Troncoso 2012 |
| CPO-RT-059 | Well | 336499 | 6969148 | 170 | -10.4 | 12/8/2009 | Troncoso 2012 |
| CPO-CE-067 | Well | 339862 | 6972842 | 176 | -10.4 | Not available | Troncoso 2012 |
| CPO-RC-36 | Well | 340406 | 6974670 | 183 | -10.3 | 1/17/2009 | Troncoso 2012 |
| CPO-CE-058 | Well | 343167 | 6975132 | 212 | -10.5 | 1/27/2010 | Troncoso 2012 |
| CPO-RT-49 | Well | 343922 | 6975800 | 191 | -10.1 | 10/13/2009 | Troncoso 2012 |
| CPO-CE-068 | Well | 344755 | 6975292 | 208 | -10.5 | 4/27/2010 | Troncoso 2012 |
| T2 | Well | 344970 | 6976250 | 210 | -10.1 | Fall 1996 | Arriagada 1997 |
| CPO-CE-065 | Well | 345950 | 6974776 | 217 | -10.5 | 4/26/2010 | Troncoso 2012 |
| CPO-RT-68 | Well | 346405 | 6976070 | 224 | -10.6 | 5/6/2010 | Troncoso 2012 |
| CPO-CE-066 | Well | 347318 | 6975609 | 218 | -10.7 | 4/26/2010 | Troncoso 2012 |
| CPO-CE-047 | Well | 347564 | 6959984 | 497 | -10.4 | 1/21/2010 | Troncoso 2012 |
| CPO-RT-48 | Well | 348506 | 6976369 | 223 | -10.6 | 10/13/2009 | Troncoso 2012 |
| CPO-CE-073 | Well | 349841 | 6978161 | 240 | -10.3 | 4/28/2010 | Troncoso 2012 |
| CPO-CE-069 | Well | 349920 | 6976204 | 226 | -10.6 | 4/27/2010 | Troncoso 2012 |
| CPO-CE-072 | Well | 350322 | 6977162 | 234 | -10.4 | 4/28/2010 | Troncoso 2012 |
| CPO-CE-056 | Well | 350929 | 6978121 | 252 | -10.3 | 1/27/2010 | Troncoso 2012 |
| CPO-CE-071 | Well | 351915 | 6977712 | 251 | -10.3 | 4/28/2010 | Troncoso 2012 |
| CPO-CE-050 | Well | 352156 | 6978811 | 275 | -10.3 | 1/22/2010 | Troncoso 2012 |
| 82 | Well | 352450 | 6977570 | 250 | -11.2 | 1/11/1996 | Arriagada 1997 |
| CPO-CE-070 | Well | 353394 | 6978717 | 255 | -10.5 | 4/28/2010 | Troncoso 2012 |
| CPO-CE-049 | Well | 354454 | 6979655 | 277 | -10.4 | 1/22/2010 | Troncoso 2012 |
| CPO-CE-055 | Well | 354560 | 6980473 | 280 | -10.2 | 1/27/2010 | Troncoso 2012 |
| CPO-RT-58 | Well | 356067 | 6979697 | 284 | -10.2 | 12/8/2009 | Troncoso 2012 |
| 81 | Well | 357420 | 6979600 | 300 | -10.5 | Summer 1996 | Arriagada 1997 |
| CPO-CE-063 | Well | 358134 | 6979652 | 282 | -9.9 | 4/24/2010 | Troncoso 2012 |
| CPO-RT-54 | Well | 358509 | 6980997 | 303 | -8.5 | 12/6/2009 | Troncoso 2012 |
| CPO-RT-70 | Well | 358592 | 6979311 | 303 | -10.0 | 7/6/2010 | Troncoso 2012 |
| CPO-CE-062 | Well | 360879 | 6978621 | 304 | -9.9 | 4/23/2010 | Troncoso 2012 |
| CPO-CE-061 | Well | 361285 | 6978382 | 310 | -10.3 | 4/23/2010 | Troncoso 2012 |
| T3 | Well | 361660 | 6977400 | 323 | -10.8 | Fall 1996 | Arriagada 1997 |
| CPO-ML-39 | Well | 361726 | 6979258 | 311 | -10.0 | 10/17/2008 | Troncoso 2012 |
| CPO-RT-50 | Well | 361771 | 6976694 | 318 | -10.5 | 10/13/2009 | Troncoso 2012 |
| CPO-ML-38 | Well | 363570 | 6976340 | 326 | -9.8 | 10/17/2008 | Troncoso 2012 |
| CPO-CE-060 | Well | 364153 | 6974378 | 344 | -10.5 | 4/21/2010 | Troncoso 2012 |
| CPO-ML-40 | Well | 364839 | 6975662 | 344 | -9.6 | 10/17/2008 | Troncoso 2012 |
| 80 | Well | 365300 | 6974920 | 340 | -10.2 | Summer 1996 | Arriagada 1997 |
| CPO-RT-52 | Well | 365464 | 6974542 | 357 | -10.4 | 12/5/2009 | Troncoso 2012 |
| CPO-CE-054 | Well | 366876 | 6972644 | 368 | -10.0 | 1/26/2010 | Troncoso 2012 |
| CPO-RT-57 | Well | 367876 | 6971860 | 384 | -10.4 | 12/7/2009 | Troncoso 2012 |
| CPO-RC-30 | Well | 368912 | 6970775 | 398 | -10.7 | 12/11/2008 | Troncoso 2012 |
| CPO-RC-85 | Well | 370006 | 6969907 | 412 | -10.4 | 9/23/2010 | Troncoso 2012 |
| CPO-RT-56 | Well | 370148 | 6970178 | 437 | -9.3 | 12/7/2009 | Troncoso 2012 |
| CPO-RC-32 | Well | 370192 | 6970146 | 416 | -10.6 | 12/17/2008 | Troncoso 2012 |
| CPO-RC-33 | Well | 370192 | 6970146 | 407 | -9.3 | 12/17/2008 | Troncoso 2012 |
| CPO-RT-51 | Well | 370953 | 6968015 | 423 | -10.5 | 12/3/2009 | Troncoso 2012 |
| CPO-RT-55 | Well | 372501 | 6967819 | 464 | -7.2 | 12/7/2009 | Troncoso 2012 |
| T5 | Well | 373200 | 6955520 | 492 | -10.3 | Fall 1996 | Arriagada 1997 |
| T4 | Well | 373560 | 6967090 | 442 | -11.1 | Fall 1996 | Arriagada 1997 |
| CPO-RT-39 | Well | 374444 | 6958601 | 499 | -10.4 | 10/8/2009 | Troncoso 2012 |
| CPO-CE-053 | Well | 374508 | 6958978 | 503 | -10.1 | 1/25/2010 | Troncoso 2012 |
| CPO-YP-50 | Well | 374550 | 6953805 | 532 | -10.2 | 12/11/2008 | Troncoso 2012 |
| CPO-RT-40 | Well | 374583 | 6965579 | 443 | -10.5 | 10/8/2009 | Troncoso 2012 |
| CPO-RT-06 | Well | 374586 | 6965585 | 443 | -10.9 | 1/19/2009 | Troncoso 2012 |
| CPO-RC-31 | Well | 374684 | 6952282 | 582 | -10.8 | 12/12/2008 | Troncoso 2012 |
| CPO-RT-28 | Well | 374773 | 6951986 | 556 | -10.5 | 10/1/2009 | Troncoso 2012 |
| CPO-YP-51 | Well | 374797 | 6951980 | 556 | -10.7 | 12/11/2008 | Troncoso 2012 |
| CPO-CE-051 | Well | 374863 | 6962559 | 451 | -10.8 | 1/23/2010 | Troncoso 2012 |
| CPO-CE-048 | Well | 374867 | 6964530 | 449 | -10.7 | 1/22/2010 | Troncoso 2012 |
| 79 | Well | 375200 | 6955425 | 500 | -10.8 | Summer 1996 | Arriagada 1997 |
| T6 | Well | 375260 | 6956070 | 525 | -11.0 | Fall 1996 | Arriagada 1997 |
| CPO-YP-53 | Well | 375752 | 6949829 | 599 | -10.8 | 12/12/2008 | Troncoso 2012 |
| 78 | Well | 376450 | 6946450 | 600 | -11.1 | Summer 1996 | Arriagada 1997 |
| CPO-RC-29 | Well | 376539 | 6945151 | 632 | -10.3 | 12/10/2008 | Troncoso 2012 |
| T9 | Well | 376725 | 6957152 | 264 | -5.6 | Winter 1996 | Arriagada 1997 |
| CPO-RT-24 | Well | 376868 | 6970720 | 505 | -6.1 | 7/9/2009 | Troncoso 2012 |
| CPO-RC-28 | Well | 376965 | 6455852 | 622 | -10.8 | 12/10/2008 | Troncoso 2012 |
| CPO-YP-52 | Well | 377110 | 6949716 | 619 | -8.6 | 12/12/2008 | Troncoso 2012 |
| CPO-YP-48 | Well | 378174 | 6943864 | 632 | -10.3 | 12/10/2008 | Troncoso 2012 |
| CPO-RT-01 | Well | 378236 | 6940558 | 640 | -10.1 | Summer 2009 | Troncoso 2012 |
| CPO-RC-77 | Well | 378775 | 6938940 | 698 | -10.4 | 12/2/2009 | Troncoso 2012 |
| CPO-YP-46 | Well | 378793 | 6938960 | 696 | -10.7 | 12/10/2008 | Troncoso 2012 |
| CPO-YP-45 | Well | 378799 | 6937759 | 712 | -10.6 | 12/9/2008 | Troncoso 2012 |
| CPO-RC-16 | Well | 379084 | 6973508 | 549 | -6.5 | 10/11/2008 | Troncoso 2012 |
| CPO-RT-30 | Well | 379085 | 6973508 | 549 | -6.4 | 10/3/2009 | Troncoso 2012 |
| CPO-RC-26 | Well | 379561 | 6936502 | 720 | -10.7 | 12/9/2008 | Troncoso 2012 |
| CPO-YP-47 | Well | 381401 | 6934973 | 733 | -10.7 | 12/10/2008 | Troncoso 2012 |
| 77 | Well | 382300 | 6931550 | 775 | -10.9 | Summer 1996 | Arriagada 1997 |
| CPO-RC-27 | Well | 382481 | 6932015 | 770 | -10.9 | 12/10/2008 | Troncoso 2012 |
| CPO-RC-25 | Well | 382548 | 6933451 | 766 | -10.4 | 12/6/2008 | Troncoso 2012 |
| CPO-YP-37 | Well | 382820 | 6932290 | 777 | -10.7 | 12/6/2008 | Troncoso 2012 |
| CPO-YP-40 | Well | 382852 | 6931274 | 771 | -11.0 | 12/8/2008 | Troncoso 2012 |
| CPO-RT-32 | Well | 382853 | 6930969 | 778 | -10.9 | 10/5/2009 | Troncoso 2012 |
| T7 | Well | 385590 | 6928150 | 820 | -11.3 | Fall 1996 | Arriagada 1997 |
| CPO-RT-37 | Well | 386970 | 6924981 | 867 | -11.0 | 10/7/2009 | Troncoso 2012 |
| CPO-RT-38 | Well | 388867 | 6923433 | 852 | -11.3 | 10/7/2009 | Troncoso 2012 |
| 76 | Surface water | 388888 | 6923500 | 900 | -10.4 | Summer 1996 | Arriagada 1997 |
| CPO-RT-27 | Well | 389740 | 6921200 | 915 | -11.1 | 10/1/2009 | Troncoso 2012 |
| CPO-RT-75 | Well | 391140 | 6920974 | 956 | -11.1 | 7/6/2010 | Troncoso 2012 |
| CPO-YP-62 | Surface water | 391630 | 6920102 | 956 | -11.0 | 5/18/2009 | Troncoso 2012 |
| CPO-RC-23 | Well | 392898 | 6918500 | 981 | -11.2 | 12/5/2008 | Troncoso 2012 |
| CPO-YP-33 | Well | 394764 | 6916888 | 996 | -11.0 | 12/4/2008 | Troncoso 2012 |
| CPO-YP-41 | Well | 397464 | 6915053 | 1004 | -11.2 | 12/9/2008 | Troncoso 2012 |
| CPO-YP-42 | Well | 397904 | 6914040 | 1023 | -11.3 | 12/9/2008 | Troncoso 2012 |
| CPO-YP-43 | Well | 398060 | 6913443 | 1031 | -11.3 | 12/9/2008 | Troncoso 2012 |
| CPO-YP-44 | Well | 398138 | 6913109 | 1031 | -11.0 | 12/9/2008 | Troncoso 2012 |
| CPO-YP-36 | Well | 398224 | 6912831 | 1047 | -11.3 | 12/6/2008 | Troncoso 2012 |
| 74 | Well | 398500 | 6912875 | 1030 | -11.7 | Summer 1996 | Arriagada 1997 |
| CPO-YP-35 | Well | 398822 | 6912179 | 1123.2 | -11.3 | 12/5/2008 | Troncoso 2012 |
| CPO-RT-44 | Surface water | 399722 | 6909301 | 1077 | -10.9 | 10/9/2009 | Troncoso 2012 |
| CPO-RT-45 | Well | 400327 | 6910245 | 1062 | -10.7 | 10/9/2009 | Troncoso 2012 |
| CPO-RC-24 | Well | 400614 | 6907334 | 1120 | -11.3 | 12/5/2008 | Troncoso 2012 |
| 73 | Well | 400788 | 6909925 | 1070 | -11.4 | Summer 1996 | Arriagada 1997 |
| CPO-RT-26 | Well | 401020 | 6905328 | 1111 | -11.6 | 9/30/2009 | Troncoso 2012 |
| 72 | Well | 401050 | 6905200 | 1110 | -11.9 | Summer 1996 | Arriagada 1997 |
| CPO-YP-32 | Well | 401056 | 6907262 | 1088 | -11.5 | 12/4/2008 | Troncoso 2012 |
| CPO-YP-30 | Well | 401102 | 6905429 | 1201.7 | -11.4 | 12/4/2008 | Troncoso 2012 |
| CPO-RT-10 | Surface water | 402376 | 6885683 | 1519 | -11.6 | Summer 2009 | Troncoso 2012 |
| CPO-RC-59 | Well | 403318 | 6892225 | 1296 | -11.7 | 5/13/2009 | Troncoso 2012 |
| CPO-RT-43 | Well | 403660 | 6894813 | 1248 | -11.6 | 10/9/2009 | Troncoso 2012 |
| CPO-RT-05 | Surface water | 403966 | 6901923 | 1163 | -11.9 | Summer 2009 | Troncoso 2012 |
| CPO-RT-42 | Well | 403999 | 6889752 | 1342 | -11.6 | 10/9/2009 | Troncoso 2012 |
| 69 | Surface water | 404250 | 6901900 | 1150 | -11.6 | Summer 1996 | Arriagada 1997 |
| 68 | Surface water | 404650 | 6889150 | 1366 | -10.5 | Summer 1996 | Arriagada 1997 |
| CPO-YP-39 | Well | 405888 | 6896949 | 1229 | -11.9 | 12/7/2008 | Troncoso 2012 |
| T8 | Well | 407000 | 6899190 | 1210 | -12.3 | Fall 1996 | Arriagada 1997 |
| CPO-RT-29 | Well | 407368 | 6898185 | 1252 | -10.9 | 10/2/2009 | Troncoso 2012 |
| CPO-YP-38 | Well | 407377 | 6898180 | 1251 | -10.8 | 12/7/2008 | Troncoso 2012 |
| CPO-ML-036 | Well | 407831 | 7003263 | 1650 | -7.0 | 10/16/2008 | Troncoso 2012 |
| 67 | Surface water | 409100 | 6900100 | 1280 | -11.2 | Summer 1996 | Arriagada 1997 |
| CPO-ML-13 | Well | 410732 | 7000674 | 1564 | -7.1 | 10/11/2008 | Troncoso 2012 |
| CPO-ML-37 | Well | 411011 | 7000546 | 1561 | -6.7 | 10/16/2008 | Troncoso 2012 |
| CPO-RT-31 | Well | 411011 | 7000546 | 1561 | -6.6 | 10/3/2009 | Troncoso 2012 |
| CPO-RT-41 | Well | 415408 | 6884128 | 1585 | -12.5 | 10/9/2009 | Troncoso 2012 |
| 66 | Surface water | 420700 | 6878800 | 1975 | -12.2 | Summer 1996 | Arriagada 1997 |
| CPO-RC-44 | Surface water | 420899 | 6881973 | 1813 | -13.1 | Summer 2009 | Troncoso 2012 |
| 70 | Surface water | 421450 | 6882000 | 1840 | -12.1 | Summer 1996 | Arriagada 1997 |
| 71 | Surface water | 422650 | 6885600 | 1990 | -11.7 | Summer 1996 | Arriagada 1997 |
| CPO-RC-50 | Surface water | 430897 | 6930327 | 2339 | -11.1 | Summer 2009 | Troncoso 2012 |
| CPO-ML-35 | Surface water | 435963 | 7015494 | 2435 | -8.2 | 10/16/2008 | Troncoso 2012 |
| 39 | Surface water | 437709 | 6878003 | 3403 | -13.0 | Summer 1996 | Arriagada 1997 |
| CPO-ML-44 | Surface water | 439996 | 6996874 | 2145 | -5.9 | 1/25/2009 | Troncoso 2012 |
| CPO-RT-21 | Surface water | 441873 | 6862681 | 4970 | -15.2 | Summer 2009 | Troncoso 2012 |
| CPO-ML-34 | Surface water | 442566 | 7017617 | 2550 | -6.4 | 10/16/2008 | Troncoso 2012 |
| CPO-RT-22 | Surface water | 442587 | 6866072 | 4284 | -12.7 | 1/23/2009 | Troncoso 2012 |
| CPO-ML-11 | Surface water | 443418 | 6999084 | 2220 | -6.0 | 9/2/2008 | Troncoso 2012 |
| CPO-RC-76 | Surface water | 443464 | 6999597 | 2250 | -6.2 | 10/6/2009 | Troncoso 2012 |
| CPO-RT-12 | Surface water | 449397 | 6900006 | 3650 | -10.9 | 1/21/2009 | Troncoso 2012 |
| CPO-RC-46 | Surface water | 451162 | 6946881 | 2896 | -8.8 | Summer 2009 | Troncoso 2012 |
| CPO-RT-20 | Surface water | 457944 | 6915363 | 3110 | -12.1 | Summer 2009 | Troncoso 2012 |
| CPO-ML-30 | Well | 463682 | 7028446 | 3247 | -5.5 | 10/15/2008 | Troncoso 2012 |
| CPO-RC-51 | Surface water | 464353 | 6937871 | 3637 | -11.2 | Summer 2009 | Troncoso 2012 |
| CPO-ML-41 | Surface water | 466923 | 7006191 | 3141 | -8.2 | 1/25/2009 | Troncoso 2012 |
| CPO-RT-35 | Well | 467500 | 7028105 | 3376 | -5.2 | 10/6/2009 | Troncoso 2012 |
| CPO-ML-29 | Well | 467548 | 7026959 | 3382 | -5.8 | 10/15/2008 | Troncoso 2012 |
| CPO-ML-31 | Well | 468433 | 7029208 | 3421 | -5.3 | 10/15/2008 | Troncoso 2012 |
| CPO-ML-42 | Surface water | 470664 | 7007552 | 3358 | -6.7 | 1/25/2009 | Troncoso 2012 |
| CPO-RT-15 | Surface water | 471604 | 6928168 | 4112 | -13.9 | 1/22/2009 | Troncoso 2012 |
| CPO-ML-22 | Well | 473211 | 6950299 | 4447 | -11.5 | 10/14/2008 | Troncoso 2012 |
| CPO-RT-16 | Well | 474025 | 6921900 | 3708 | -13.0 | 1/22/2009 | Troncoso 2012 |
| CPO-RT-46 | Well | 474025 | 6921900 | 3708 | -12.7 | 10/11/2009 | Troncoso 2012 |
| CPO-RT-14 | Surface water | 474705 | 6905325 | 3305 | -12.0 | Summer 2009 | Troncoso 2012 |
| CPO-RT-34 | Well | 477020 | 7038260 | 4244 | -7.6 | 10/6/2009 | Troncoso 2012 |
| CPO-ML-32 | Well | 477532 | 7039241 | 4278 | -9.1 | 10/15/2008 | Troncoso 2012 |
| CPO-RT-33 | Well | 477532 | 7039241 | 4278 | -9.1 | 10/6/2009 | Troncoso 2012 |
| CPO-ML-33 | Surface water | 477703 | 7029005 | 3825 | -5.5 | 10/16/2008 | Troncoso 2012 |
| CPO-ML-27 | Well | 491277 | 7007668 | 3818 | -10.2 | 10/15/2008 | Troncoso 2012 |
